# SGLT2 Inhibition in Acute Myocardial Infarction—A Comprehensive Review

**DOI:** 10.31083/j.rcm2402032

**Published:** 2023-01-31

**Authors:** Martin Benedikt, Ewald Kolesnik, Harald Sourij, Dirk von Lewinski

**Affiliations:** ^1^Division of Cardiology, Department of Internal Medicine, Medical University of Graz, 8010 Graz, Austria; ^2^Division of Endocrinology and Diabetology, Interdisciplinary Metabolic Medicine Trials Unit, Department of Internal Medicine, Medical University of Graz, 8010 Graz, Austria

**Keywords:** SGLT2, myocardial infarction, metabolism, inflammation

## Abstract

In heart failure as well as in chronic kidney disease sodium-glucose cotransporter 2 (SGLT2) inhibitors have changed the landscape of medical therapy. Originally developed for use in diabetes, an unforeseen cardiovascular benefit extended SGLT2 inhibitor use from antihyperglycemic agents to cardiovascular and renal risk modifying agents. As their benefit in cardiovascular disease is independent from the diabetic state as well as the left ventricular ejection fraction it is the only class of therapy recommended throughout the spectrum of heart failure. Until very recently, the remaining gap in evidence has been data on the safety and efficacy of SGLT2 inhibitors in patients with acute myocardial infarction (MI) as former trials of SGLT2 inhibitors to date have excluded patients with recent ischemic events. As the first out of three trials conducted in post MI SGLT2 inhibitors therapy the EMMY trial was published. EMMY randomized 476 patients shortly after percutaneous intervention for recent large MI to either 10 mg of empagliflozin daily or placebo. The primary endpoint of changes in N-terminal pro brain natriuretic peptide (NT-proBNP) over 26 weeks as well as the functional and structural secondary endpoints were met. This provides first evidence of SGLT2 inhibitors-mediated beneficial results in this group of patients. We here discuss these results in the light of the two upcoming outcome trials (DAPA-MI and EMPACT-MI) with regard to the future role of this class of drugs early after MI.

## 1. Development of Post-MI Therapy

The pathophysiology of myocardial infarction is a complex mechanism. It includes acute myocardial ischemia driven by energy depletion followed by early reperfusion injury developing during the first minutes/hours of reperfusion, and the subsequent remodeling phase during the first days/weeks after myocardial infarction (MI) resulting in an irreversible necrotic damage of the concerning area. Also, the neurohumoral system is involved with initiation of the renin-angiotensin-aldosterone system and upregulation of the sympathetic nervous system. These mechanisms lead to structural deterioration in the myocardium, called remodelling, affecting functional processes in ischemic and non-ischemic areas. However, effective clinical therapy against reperfusion injury is still missing, despite numerous therapies being effective in preclinical models [1, 2, 3]. In this review we report on the use of sodium-glucose cotransporter 2 (SGLT2) inhibitors’s to combat the remodeling phase of myocardial infarction.

In the past, therapeutic options have been established for the acute treatment of post-myocardial infarction aiming to reduce myocardial damage in the acute setting as well as to reduce further functional and structural changes. Early initiation of these substances after myocardial infarction has shown beneficial effects on mortality and major adverse cardiovascular events (MACE). First, the SAVE-trial with captopril showed a relative risk reduction of 19% for mortality in patients after myocardial infarction and left ventricular dysfunction compared to placebo. In addition, the trial showed a decrease in the incidence of both fatal and nonfatal major cardiovascular events with a relative risk reduction of 21% for death from cardiovascular causes, 37% for the development of severe heart failure and 25% for recurrent myocardial infarction [4]. Similarly, trandolapril (TRACE-trial) and ramipril (AIRE-trial) improved outcome if initiated early after acute myocardial infarction [5, 6]. A comprehensive meta-analysis of large, randomized placebo-controlled trials including ~100,000 patients highlighted beneficial effects of angiotensin converting enzyme (ACE)-inhibitors on long-term survival with 85% of the effect within the first seven days after myocardial infarction [7]. In line, two large clinical trials were showing similar results for the angiotensin-receptor blocker valsartan in the VALIANT trail [8] and losartan in the OPTIMAAL trial [9]. Comparable beneficial results were also reported for betablockers. Metoprolol reduced the all-cause mortality, cardiovascular mortality, ventricular fibrillation, and non-fatal myocardial infarction in patient post myocardial infarction and left ventricular systolic dysfunction compared to placebo [10, 11]. However, it has to be acknowledged that this therapy can also increase the risk of developing cardiogenic shock because of its negative chronotropic and inotropic effects [11] and therefore should be avoided in hypotonic or bradycardic patients.

Overall, ACE-inhibitors/angiotensin-receptor blocker as well as betablockers should be early initiated in the acute setting of acute myocardial infarction [12, 13, 14].

The additional treatment with the mineralocorticoid receptor antagonist eplerenone resulted in reductions in mortality and the rate of death from cardiovascular causes or hospitalization for cardiovascular events in the acute setting of myocardial infarction complicated by left ventricular dysfunction and heart failure in the EPHESUS-trial. In addition, there was also a reduction in cardiovascular mortality and the rate of death from any cause or any hospitalization in the eplerenone group compared to placebo [15]. For spironolactone similar results were observed for the STEMI-subgroup, but no beneficial effects were noted for the early initiation in the NSTEMI-subgroup compared to placebo in the ALBATROSS-trial [16].

In contrast, the angiotensin-neprilysin inhibitor sacubitril/valsartan has been reported to show a significant reduction of cardiovascular death and hospitalization for heart failure in heart failure with reduced ejection fraction (HFrEF) compared to enalapril in the PARADIGM-trial [17] and led to a greater reduction in the N-terminal pro brain natriuretic peptide (NT-proBNP) concentration than enalapril in acute decompensated heart failure in the PIONEER-trial [18]. However, patients with acute myocardial infarction and reduced left ventricular ejection fraction in the PARADISE-MI trial did not show significantly lower incidence of death from cardiovascular causes or incident heart failure compared to ramipril [19]. Interestingly, the very large subgroup of patients treated with percutaeous coronary intervention (PCI) as recommended in the guidelines and accounting for 88% of the total trial population did have a significantly better outcome.

With respect to SGLT2 inhibition there is no clinical data available on outcome after acute myocardial infarction yet. The small EMBODY trial reported effects of empagliflozin initiated shortly after the acute phase focusing on sympathomimetic activity [20].

Two ongoing outcome trials are testing the effects of dapagliflozin vs. placebo (DAPA-MI) on the composite endpoint cardiovascular death and hospitalization for heart failure and of empagliflozin vs. placebo (EMPACT-MI) on the composite endpoint time to first heart failure hospitalisation or all-cause mortality. Longitudinal data on functional and structural parameters after SGLT2 inhibitor use is scarce and no data available in post MI patients. The recently published EMMY-trial [21] now filled that gap being the first trial showing a significant reduction in the primary endpoint NT-proBNP as well as functional and structural echo parameters using the SGLT2-inhibitor empagliflozin compared to placebo and thus represents a new treatment option in patients with acute myocardial infarction.

## 2. The Role of SGLT2 Inhibitors in Myocardial Infarction

The SGLT2-receptor is mainly expressed in the proximal tube of human nephrons and intestinal cells and thus play an important role in renal glucose excretion and lowering blood glucose levels [22] but could not be detected in human myocardium so far [23]. Thus, there is no receptor-mediated effect of SGLT2-inhibitors on the human myocardium, but various indirect pathways seem to play an important role in the anti-remodeling effects [24]. A key role of the SGLT2 pathway is an increased myocardial uptake of ketone bodies resulting in an improved myocardial energy supply and thus impacts the energetic state of the myocardial cells leading to a decrease in cardiac necrosis and cardiac dysfunction [24]. Moreover, various experimental studies showed potential beneficial myocardial effects of SGLT2-inhibition through upregulation of cardioprotective proteins. Asensio *et al*. [25] published an increased uptake of the GTP enzyme cyclohydrolase-1 for empagliflozin independent of diabetic status affecting the cGCH1-BH4/NO-pathway and resulting in a reduction of cardiac dysfunction by its anti-remodeling effect. Next, SGLT2-inhibitors demonstrates positive cardiac effects in decreasing cytoplasmatic sodium through inhibition of the Na+/H+ exchanger sodium-hydrogen antiporter 1 (NHE1) with alternations in cellular calcium hemostasis by intracellular calcium overload in rats and rabbits [26] as well as in mice [27], although the effects on the NHE1 were independent of the SGLT-receptor [26, 27]. SGLT2-inhibitors ameliorates left ventricular remodeling in heart failure by the adenosine monophosphate-activated protein kinase (AMPK)-pathway with reduction in myocardial necrosis and cardiac inflammatory processes [28] through enhancing myocardial energetics [29] and attenuating ischemia and reperfusion injury [30]. A further mechanism of SGLT2-inhibition is the reduction of oxidate stress levels mediating anti-inflammatory effects by the BCL2- as well as Signal transducer and activator of transcription 3/janus kinase 2 (STAT3/JAK2)-pathway and thus, decreasing myocardial necrosis and cardiac dysfunction [31, 32, 33]. All these preclinical molecular effects ameliorates inflammatory response and necrosis of cardiomyocytes resulting in smaller infarct size, less reperfusion injury and anti-remodeling effects.

First trial to show the effects of SGLT2-inhibitors in acute myocardial infarction was by Paolisso *et al*. [34] reporting a significant decrease in initial inflammatory parameters (white-blood-cell count, neutrophil-to-lymphocyte ratio (NLR), platelet-to-lymphocyte ratio (PLR), neutrophil-to-platelet ratio (NPR), and C-reactive protein) as well as infarct size (echocardiographic parameters and peak troponin levels) in diabetic patients already treated with an SGLT2-inhibitor before myocardial infarction compared to other oral anti-diabetic agents independently of glucose-metabolic control. Based on this trial, it can be speculated that potential anti-inflammatory effects of SGLT2-inhibitors after acute myocardial infarction mediate beneficial cardiovascular outcome such as reducing infarct size and anti-remodeling effects. In addition, a diuretic effect with impact on hemodynamics via lowering the blood pressure as well as an increase in hematocrit have been described in the EMPA-REG OUTCOME trial [35, 36, 37]. The antiarrhythmic properties of SGLT2-inhibitors in patients with structural and functional cardiac diseases is still unclear. First trial to show strong evidence with beneficial effects was the DAPA-HF demonstrating in a post-hoc analysis a significant lower number of serious ventricular arrythmias, resuscitated cardiac arrest and sudden death in patients with HFrEF with a relative risk reduction of 21% in dapagliflozin compared to placebo [38]. Regarding atrial fibrillation a post hoc analysis of the DELACRE-TIMI 58 trial attenuates the incidence of atrial fibrillation with a relative risk reduction of 19% in dapagliflozin compared to placebo in patients with type 2 diabetes mellitus (T2DM) [39]. Further, two recent metanalysis including large clinical trials show a significant reduction in the incidence of atrial fibrillation in patients treated with an SGLT2-inhibitors [40, 41]. Based on these finding, further trials need to be initiated to observe potential effects of SGLT2-inhibitors on arrhythmic events. A recent ongoing study, the ERASe-trial, is a placebo-controlled randomized investigator initiated phase 3b trial in Austria proving potential beneficial effect of the SGLT2-inhibitor ertugliflozin on ventricular arrhythmias in patients with internal cardioverter defibrillator (ICD) ± cardiac resynchronisation therapy (CRT) [42]. Indirect data on SGLT2 inhibitors with respect to acute myocardial infarction was derived from a meta-analysis of the EMPA-REG OUTCOME-, CANVAS and DECLARE TIMI-58 trials depicting a reduction of 11% in major adverse cardiovascular events defined as MI, stroke, and cardiovascular death (hazard ratio (HR) 0.89 [95% cofidence interval (CI) 0.83 to 0.96], *p* = 0.0014). Benefits observed in patients with manifest coronary artery disease tended to be even larger with a reduction of 15% (HR 0.86 [0.80–0.93] *p* for interaction = 0.0501) for SGLT2-inhibitors vs. placebo [43]. A recent meta-analysis of 238 clinical randomized trials showed a reduction of cardiovascular mortality in patients with SGLT-2 inhibitors compared to placebo with an odds ratio of 0.84 (95% CI 0.76 to 0.92) and a reduction in non-fatal myocardial infarction with an odds ratio 0.87 (95% CI 0.79 to 0.97) [44].

## 3. EMMY as the First SGLT2-Inhibitor Trial in Post MI Treatment

SGLT2-inihibtors have shown various beneficial effects in patients with chronic heart failure, diabetes as well as chronic kidney disease, but data in the setting of an acute myocardial infarction is scarce.

The EMMY-trial is the first multicenter, randomized, double-blind, placebo-controlled trial assessing the impact of empagliflozin on biomarkers of heart failure as well as cardiac function and structure in patients with acute myocardial infarction. It is the first clinical trial to show beneficial effects of SGLT2-inhibitors in patients with an acute myocardial infarction compared to placebo [45] and was recently published in the European Heart Journal [21].

From 11th May 2017 until 3rd May 2022, eleven Austrian centers enrolled 476 patients with large acute myocardial infarction (defined as creatinin kinase (CK) >800 and troponin >10× upper limit of normal (ULN)) receiving 10 mg empagliflozin once daily vs. placebo in addition to guideline directed medical therapy within 72 hours after percutaneous coronary intervention.

Participants had a median age (IQR) of 57 (52–64) years, an average BMI of 27.6 (25.1–30.3) kg/$m^{2}$ with 18% of patients were female and 13% of study participants had established type 2 diabetes. Median baseline CK (IQR) for measuring infarct size was 1673 U/l (1202–2456), the median (IQR) NT-proBNP at baseline was 1294 pg/mL (757–2246) and the median systolic pressure was 125 mmHg (117–131). More than 96% of the patients received state-of-the-art post-MI medical therapy consisting of ACEI/Angiotensin receptor blocker (ARB)/angiotensin receptor-neprilysin inhibitor (ARNI), betablocker, and Statin. 40% of all patients received an mineral corticoid antagonist (MRA).

The primary outcome, being the change in NT-proBNP levels, was 15% (95% CI –4.4 to –23.6%) lower with empagliflozin at week 26 compared to placebo (*p* = 0.026). This larger decline could already be observed after 12 weeks (–13.3%, 95% CI –3.0 to –22.5%) of treatment (*p* = 0.021).

Secondary outcomes included changes in echocardiographic parameters, HbA1c, body weight, and ketone bodies. The circulating ketone bodies concentrations (β-hydroxybutyric acid) was 0.123 ± 0.021 and 0.0917 ± 0.01 mmol/L in the empagliflozin group and the placebo group at baseline, respectively. It showed a significant increase in the empagliflozin group (Δ = 41.9%; 95% CI 21.8 to 63.8%, *p *< 0.0001) compared to the place group (Δ = 23.4%; 95% CI 5.9–42.4%, *p* = 0.0066).

The trial showed a significantly larger increase of left ventricular function (by absolute 1.5%; 95% CI 0.15 to 2.88%; *p* = 0.029) in the empagliflozin as compared to the placebo group and a significantly better development of diastolic function in the empagliflozin group with an E/e’ at week 26 being 6.8% (95% CI 1.3% to 11.3%; *p* = 0.015) lower than placebo. Structural parameters were also significantly improved with left ventricular endsystolic and end-diastolic volumes being significantly lower in the empagliflozin group (see Table 1).

**Table 1.** **Primary and secondary echocardiography outcome parameters**.

	Empagliflozin				Placebo				
	Baseline	Week 26	Absolute change	Percent change	Baseline	Week 26	Absolute change	Percent change	Difference at week 26
LV-EF (%)	48 (43–53)	53 (47–58)	4.7 (3.6; 5.8)	11.1 (8.6; 13.6)	49 (43–54)	52 (47–57)	2.8 (1.8; 3.9)	7.6 (5.2; 9.9)	1.5% (95% CI 0.2% to 2.9%; *p* = 0.029)
E/e’	8 (7–11)	8 (7–9)	–1.3 (–1.6; –0.9)	–9.7 (–13.1; –6.4)	9 (8–11)	8 (7–10)	–0.7 (–1.1; –0.4)	–3.5 (–7.4; 0.4)	–6.8% (95% CI –11.3% to –1.3%; *p* = 0.015)
LVESV (mL)	61 (48–76)	60 (46–73)	–3.6 (–6.3; –1.0)	–2.2 (–6.4; 2.0)	60 (46–73)	59 (46–81)	4.30 (1.2; 7.4)	12.1 (6.4; 17.7)	–7.5 mL (95% CI –11.5 to –3.4; *p* = 0.0003)
LVEDV (mL)	119 (93–139)	122 (101–145)	3.4 (–0.7; 7.4)	5.9 (1.8; 10.1)	114 (92–134)	120 (100–154)	13.5 (8.7; 18.3)	14.8 (10.2; 19.4)	–9.7 mL (95% CI –15.7 to –3.7; *p* = 0.001)

LV-EF, left ventricular ejection fraction; LVESV, left ventricular end systolic volume; LVEDV, left ventricular end diastolic volume; CI, Confidence interval.

Moreover, with empagliflozin we observed the body weight to be significantly more decreased than in the placebo group and ketone bodies were significantly increased after 26 weeks of treatment. Of note, no difference in HbA1c levels was observed as only a minority (13%) of patients suffered from type 2 diabetes.

According to the adverse events, no amputation, no ketoacidotic events, and no severe hypoglycemia were reported. Only seven patients were hospitalized for heart failure (3 in the placebo group and 4 in the empagliflozin group). Three patients died (all in the empagliflozin group), one due to lung cancer and two of cardiogenic shock due to large myocardial infarction size.

Based on these results, the EMMY trial is the first clinical trial to show beneficial effects of SGLT2-inhibitors on biomarkers and cardiac function and structure in patients with acute myocardial infarction and enables to speculate on beneficial outcomes of the upcoming large outcome trials DAPA-MI and EMPACT-MI.

## 4. Upcoming Outcome Trials in Post MI Patients

DAPA-MI and EMPACT-MI are investigating the effect of dapagliflozin and empagliflozin, respectively on the incidence of heart failure or cardiovascular (or all-cause in EMPACT-MI) death in patients with acute myocardial infarction, respectively. DAPA-MI is a registry-based RCT whereas EMPACT-MI is a traditional RCT.

Table 2 summarizes sample sizes, inclusion and exclusion criteria as well as outcome measures for these trials in comparison to the EMMY trial. As shown, EMMY focused on heart failure biomarker as well as functional and structural changes in the heart, whereas DAPA-MI and EMPACT-MI use typical heart failure endpoints. Moreover, EMMY on the one side recruited MI patients independent of heart failure signs, symptoms or enrichment factors whereas DAPA-MI and EMPACT-MI require additional heart failure associated factors. Lastly, due to their more than 10-fold larger size DAPA-MI and EMPACT-MI are powered for hard clinical endpoints whereas EMMY was originally designed for changes in NT-proBNP and echocardiography parameters only.

**Table 2.** **Current trials with SGLT2 inhibitor treatment post myocardial infarction**.

	EMMY (Phase III)	EMPACT-MI (Phase III)	DAPA-MI (Phase III)
Drug	Empagliflozin vs. placebo	Empagliflozin vs. placebo	Dapagliflozin vs. placebo
Trial design	RCT	RCT	Registry-based RCT
Number of patients	476 patients	6500 patients	6400 patients
Primary endpoint	Change of NT-proBNP	Composite of time to first HHF or all-cause mortality	Composite of time to first occurrence of HHF or CV death
Randomization	Within 3 days after MI	Within 14 days after MI	Within 10 days after MI
Age	18–80 years of age	≥18 years of age	≥18 years of age
Kidney function	eGFR >45 mL/min/1.73 $m^{2}$	eGFR >20 mL/min/1.73 $m^{2}$	eGFR >20 mL/min/1.73 $m^{2}$
Follow-up period	26 weeks	26 months	Up to approximately 3 years
Centres	11 centres	465 centres	113 centres
Start date	May 11, 2017	December 16, 2020	December 22, 2020
Status	Completed	Ongoing	Ongoing
Inclusion criteria	- Large MI (STEMI & NSTEMI) with CK >800 U/L	- MI (STEMI & NSTEMI)	- MI (STEMI & NSTEMI)
	- BP >110/70 mmHg	- High risk of HF, defined as EITHER	- Either impaired LV systolic function or evidence of Q wave MI
		- Symptoms or signs of congestion that require treatment	- Hemodynamically stable
		OR	
		- Newly developed LVEF	
		<45%	
		AND	
		- At least one further CV risk factor	
Exclusion criteria	- Any other form of diabetes mellitus than type 2 diabetes mellitus, history of diabetic ketoacidosis	- Type I diabetes mellitus	- Known T1DM or T2DM history of ketoacidosis
	- Blood pH <7.32	- Diagnosis of chronic HF	- Chronic symptomatic HF with a prior HHF within the last year and known reduced ejection fraction (LVEF ≤40%)
	- Known allergy to SGLT-2 inhibitors	- Systolic blood pressure <90 mmHg	- Severe hepatic impairment (Child-Pugh class C)
	- Hemodynamic instability	- Cardiogenic shock or use of i.v. inotropes in last 24 hours before randomisation	- Active malignancy requiring treatment
	- >1 episode of severe hypoglycemia within the last 6 months	- Coronary Artery Bypass Grafting planned	
	- Females of childbearing potential	- Current diagnosis of Takotsubo cardiomyopathy	
	- Acute symptomatic UTI or genital infection	- Any current severe valvular heart disease	

Abbreviations: RCT, randomized controlled trial; NT-proBNP, N-terminal pro brain natriuretic peptide; HHF, hospitaisation for heart failure; CV, cardio-vascular; MI, myocardial infarction; eGFR, estimated glomerular filtration rate; STEMI, ST elevation myocardial infraction; NSTEMI, non-ST elevation myocardial infraction; CK, creatinin kinase; BP, blood pressure; LV, left ventricular; T1DM, type 1 diabetes mellitus; T2DM, type 2 diabetes mellitus; LVEF, left ventricular ejection fraction; HF, heart failure; UTI, urinary tract infection; EMMY, Empagliflozin in acute myocardial infarction; EMPACT-MI, A Streamlined, Multicentre, Randomised, Parallel Group, Double-blind Placebo-controlled Superiority Trial to Evaluate the Effect of EMPAgliflozin on Hospitalisation for Heart Failure and Mortality in Patients With aCuTe Myocardial Infarction; DAPA-MI, Dapagliflozin Effects on Cardiovascular Events in Patients With an Acute Heart Attack; SGLT-2, sodium glucose co-transporter type 2.

## 5. New Directions of Medical Therapy after MI

Heart failure is a major complication following severe acute myocardial infarction and develops in about 17% of all patients with acute myocardial infarction (AMI) [46]. Many pharmacological options have shown to have significant beneficial effects in reducing long-term cardiovascular outcome (ACEi, ARBs, ARNI, MRA, Betablocker (BB)). Since publishing the results of the EMPA-REG OUTCOME trial, beneficial effects of SGLT2-inhibitors on hospitalization for heart failure and potentially mortality became obvious in diabetic patients. Almost identical beneficial effects were reported later for patients with heart failure with reduced, mildly reduced and preserved ejection fraction for empagliflozin (EMPEROR-reduced, EMPEROR preserved) as well as dapagliflozin (DAPA-HF, DELIVER) independent of the glycemic state. This led to the implementation in recent guidelines for the treatment of chronic heart failure [14, 47]. However, beneficial effects of SGLT2 inhibition after myocardial infarction might reach beyond improving heart failure.

The EMMY trial was positive with respect to its primary endpoint (change in NTproBNP) although this effect was only moderate. NT-proBNP has been shown to be an established parameter for neurohumoral activation and a powerful predictor for cardiovascular prognosis in patients with AMI within 180 days [48, 49, 50]. A significant decline in NT-proBNP levels in patients suffering from large AMI was associated with subsequent cardiovascular outcome [49, 50]. However, changes in NT-proBNP were only mildly or even non-significantly affected in recent SGLT2 inhibitor trials in heart failure patients [51, 52, 53]. In the randomized, double-blind, placebo-controlled multicentre EMPA-RESPONSE-AHF trial the SGLT2-inhibitor empagliflozin had no beneficial effect on dyspnoe score, weight, NTproBNP levels within the observation period of up to day 4 after the index event as well as length of hospital stay but showed a reduced combined endpoint of worsening HF, rehospitalization for HF or death at 60 days and a significant increase in urinary output compared to placebo [54]. Moreover, absolute NT-proBNP concentrations did not significantly differ in EMPEROR-Preserved when analyzed after 52 weeks [55], but a recent analysis depicted significantly lower NT-proBNP concentrations in the empagliflozin group after 52 weeks when adjusted geometric mean NT-proBNP values were used for statistical analysis [56]. Interestingly, this modest NT-proBNP difference was associated with a highly significant improvement in clinical outcome. Similarly, a retrospective analysis of the CANVAS trial attributed only ~10% of the reduction in hospitalization due to heart failure in this trial to the NT-proBNP lowering seen with canagliflozin [51]. This view is supported by recent meta-analyses showing highly significant reductions in hospitalization for heart failure for HFrEF [57] and for HFpEF [58, 59, 60] despite only moderate and/or non-significant larger NT-proBNP reductions with SGLT2 inhibitor treatment.

The trial demonstrated a significant increase in ketone bodies after 26 weeks in empagliflozin compared to placebo. Given the fact that circulating ketone bodies are increased after acute myocardial infarction, higher ketone bodies are associated with functional outcome after STEMIs [61]. Upregulation of ketone bodies mediates a higher utilization resulting in increased energy supply with reduction of myocardial necrosis and cardiac dysfunction due to its anti-remodeling effects and, thus suggest a potential role for ketone metabolism in response to myocardial ischemia [24]. However, increased blood ketones are not always observed with SGLT2 inhibitors’s in preclinical [62] and clinical research [63, 64] and therefore, measurement of circulating ketone bodies and their trajectories after an index event as well as additional biomarkers will certainly play an important role in further clinical research of cardiac diseases.

Although, the EMMY trial shows significant reduction of NT-proBNP levels in acute myocardial infarction compared to placebo, the trial was not powered for hard clinical endpoints like hospitalization for heart failure or mortality. It has also to be noted that these clinical endpoints were rather infrequent in the EMMY trial. Despite the differences between EMMY and the two upcoming outcome trials with respect to more heart failure enriched populations and longer follow-up periods in the two latter ones, one might anticipate rather low event rates in the large trials, too. This already led to the increases in the enrollment target of the EMPACT-MI trial twice, now planning to recruit 6500 patients instead of the originally planned 3312 participants.

Based on the EMMY endpoints and previous outcome trials [55, 65, 66, 67] outcome numbers are likely to be mainly based on heart failure events rather than mortality in DAPA-MI and EMPACT-MI as well.

The degree of LVEF recovery after MI is an important prognostic factor and patients showing no recovery in LVEF after MI are at high risk of sudden cardiac arrest events and death [68]. Moreover, LVEF recovery was associated with a substantial decrease in all-cause and cardiovascular mortality [69]. Concerning systolic function, the EMMY-trial shows a greater increase in LV-EF in the empagliflozin group compared to placebo post MI.

Furthermore, the EMMY-trial showed an improvement in diastolic function in line with data on empagliflozin being the first pharmacological treatment option in patients with HFpEF improving long-term clinical outcome [55].

## 6. Conclusions

With the recently published EMMY-trial, the first randomized and placebo-controlled trial is available showing the efficacy of empagliflozin on cardiac biomarkers as well as cardiac function and structure post MI independent. This is accompanied by a placebo-like safety profile. As the trial was not powered for hard clinical endpoints due to comparably low patient numbers and a rather short follow-up period of 26 weeks, NT-proBNP was selected as surrogate parameter for primary outcome. The lower NT-proBNP levels in the empagliflozin group together with functional (LV-EF, diastolic function) and structural (LVESV, LVEDV) benefits, the EMMY trial provides evidence for a clinical benefit of SGLT2-inhibitors in patients suffering from MI. Based on the finding of large clinical trials and meta-analyses, NT-proBNP represents an effective outcome parameter for SGLT2 inhibition in heart failure, but potential clinical effects of SGLT2-inhibitors seem to be underestimated.

## References

[1] Lecour S, Andreadou I, Bøtker HE, Davidson SM, Heusch G, Ruiz-Meana M (2021). IMproving Preclinical Assessment of Cardioprotective Therapies (IMPACT) criteria: guidelines of the EU-CARDIOPROTECTION COST Action. *Basic Research in Cardiology*.

[2] Heusch G (2017). Critical Issues for the Translation of Cardioprotection. *Circulation Research*.

[3] Hausenloy DJ, Yellon DM (2013). Myocardial ischemia-reperfusion injury: a neglected therapeutic target. *Journal of Clinical Investigation*.

[4] Pfeffer MA, Braunwald E, Moyé LA, Basta L, Brown EJ, Cuddy TE (1992). Effect of Captopril on Mortality and Morbidity in Patients with Left Ventricular Dysfunction after Myocardial Infarction. Results of the survival and ventricular enlargement trial. *New England Journal of Medicine*.

[5] Køber L, Torp-Pedersen C, Carlsen JE, Bagger H, Eliasen P, Lyngborg K (1995). A Clinical Trial of the Angiotensin-Converting–Enzyme Inhibitor Trandolapril in Patients with Left Ventricular Dysfunction after Myocardial Infarction. *New England Journal of Medicine*.

[6] (1993). Effect of ramipril on mortality and morbidity of survivors of acute myocardial infarction with clinical evidence of heart failure. The Acute Infarction Ramipril Efficacy (AIRE) Study Investigators. *Lancet (London, England)*.

[7] (1998). Indications for ACE inhibitors in the early treatment of acute myocardial infarction: systematic overview of individual data from 100,000 patients in randomized trials. ACE Inhibitor Myocardial Infarction Collaborative Group. *Circulation*.

[8] Pfeffer MA, McMurray JJV, Velazquez EJ, Rouleau J, Køber L, Maggioni AP (2003). Valsartan, Captopril, or both in Myocardial Infarction Complicated by Heart Failure, Left Ventricular Dysfunction, or both. *New England Journal of Medicine*.

[9] Dickstein K, Kjekshus J (2002). Effects of losartan and captopril on mortality and morbidity in high-risk patients after acute myocardial infarction: the OPTIMAAL randomised trial. Optimal Trial in Myocardial Infarction with Angiotensin II Antagonist Losartan. *Lancet*.

[10] Dargie HJ (2001). Effect of carvedilol on outcome after myocardial infarction in patients with left-ventricular dysfunction: the CAPRICORN randomised trial. *Lancet*.

[11] Chen ZM, Pan HC, Chen YP, Peto R, Collins R, Jiang LX (2005). Early intravenous then oral metoprolol in 45,852 patients with acute myocardial infarction: randomised placebo-controlled trial. *Lancet*.

[12] Ibanez B, James S, Agewall S, Antunes MJ, Bucciarelli-Ducci C, Bueno H (2018). 2017 ESC Guidelines for the management of acute myocardial infarction in patients presenting with ST-segment elevation. *European Heart Journal*.

[13] Collet J-P, Thiele H, Barbato E, Barthélémy O, Bauersachs J, Bhatt DL (2021). 2020 ESC Guidelines for the management of acute coronary syndromes in patients presenting without persistent ST-segment elevation. *European Heart Journal*.

[14] McDonagh TA, Metra M, Adamo M, Gardner RS, Baumbach A, Böhm M (2021). 2021 ESC Guidelines for the diagnosis and treatment of acute and chronic heart failure. *European Heart Journal*.

[15] Pitt B, Remme W, Zannad F, Neaton J, Martinez F, Roniker B (2003). Eplerenone, a Selective Aldosterone Blocker, in Patients with Left Ventricular Dysfunction after Myocardial Infarction. *New England Journal of Medicine*.

[16] Beygui F, Cayla G, Roule V, Roubille F, Delarche N, Silvain J (2016). Early Aldosterone Blockade in Acute Myocardial Infarction: The ALBATROSS Randomized Clinical Trial. *Journal of the American College of Cardiology*.

[17] McMurray JJV, Packer M, Desai AS, Gong J, Lefkowitz MP, Rizkala AR (2014). Angiotensin–Neprilysin Inhibition versus Enalapril in Heart Failure. *New England Journal of Medicine*.

[18] Velazquez EJ, Morrow DA, DeVore AD, Duffy CI, Ambrosy AP, McCague K (2019). Angiotensin–Neprilysin Inhibition in Acute Decompensated Heart Failure. *New England Journal of Medicine*.

[19] Pfeffer MA, Claggett B, Lewis EF, Granger CB, Køber L, Maggioni AP (2021). Angiotensin Receptor-Neprilysin Inhibition in Acute Myocardial Infarction. *The New England Journal of Medicine*.

[20] Shimizu W, Kubota Y, Hoshika Y, Mozawa K, Tara S, Tokita Y (2020). Effects of empagliflozin versus placebo on cardiac sympathetic activity in acute myocardial infarction patients with type 2 diabetes mellitus: the EMBODY trial. *Cardiovascular Diabetology*.

[21] von Lewinski D, Kolesnik E, Tripolt NJ, Pferschy PN, Benedikt M, Wallner M (2022). Empagliflozin in acute myocardial infarction: the EMMY trial. *European Heart Journal*.

[22] Kosiborod M, Birkeland KI, Cavender MA, Fu AZ, Wilding JP, Khunti K (2018). Rates of myocardial infarction and stroke in patients initiating treatment with SGLT2-inhibitors versus other glucose-lowering agents in real-world clinical practice: Results from the CVD-REAL study. *Diabetes, Obesity and Metabolism*.

[23] Di Franco A, Cantini G, Tani A, Coppini R, Zecchi-Orlandini S, Raimondi L (2017). Sodium-dependent glucose transporters (SGLT) in human ischemic heart: a new potential pharmacological target. *International Journal of Cardiology*.

[24] von Lewinski D, Benedikt M, Tripolt N, Wallner M, Sourij H, Kolesnik E (2021). Can sodium glucose cotransporter 2 (SGLT-2) inhibitors be beneficial in patients with acute myocardial infarction. *Kardiologia Polska*.

[25] Asensio Lopez MDC, Lax A, Hernandez Vicente A, Saura Guillen E, Hernandez-Martinez A, Fernandez del Palacio MJ (2020). Empagliflozin improves post-infarction cardiac remodeling through GTP enzyme cyclohydrolase 1 and irrespective of diabetes status. *Scientific Reports*.

[26] Baartscheer A, Schumacher CA, Wüst RCI, Fiolet JWT, Stienen GJM, Coronel R (2017). Empagliflozin decreases myocardial cytoplasmic Na+ through inhibition of the cardiac Na+/H+ exchanger in rats and rabbits. *Diabetologia*.

[27] Uthman L, Baartscheer A, Bleijlevens B, Schumacher CA, Fiolet JWT, Koeman A (2018). Class effects of SGLT2 inhibitors in mouse cardiomyocytes and hearts: inhibition of Na+/H+ exchanger, lowering of cytosolic Na+ and vasodilation. *Diabetologia*.

[28] Koyani CN, Plastira I, Sourij H, Hallström S, Schmidt A, Rainer PP (2020). Empagliflozin protects heart from inflammation and energy depletion via AMPK activation. *Pharmacological Research*.

[29] Santos-Gallego CG, Requena-Ibanez JA, San Antonio R, Ishikawa K, Watanabe S, Picatoste B (2019). Empagliflozin Ameliorates Adverse Left Ventricular Remodeling in Nondiabetic Heart Failure by Enhancing Myocardial Energetics. *Journal of the American College of Cardiology*.

[30] Zhou X, Jin M, Liu L, Yu Z, Lu X, Zhang H (2020). Trimethylamine N-oxide and cardiovascular outcomes in patients with chronic heart failure after myocardial infarction. *ESC Heart Failure*.

[31] Andreadou I, Efentakis P, Balafas E, Togliatto G, Davos CH, Varela A (2017). Empagliflozin Limits Myocardial Infarction in Vivo and Cell Death in Vitro: Role of STAT3, Mitochondria, and Redox Aspects. *Frontiers in Physiology*.

[32] Lahnwong S, Palee S, Apaijai N, Sriwichaiin S, Kerdphoo S, Jaiwongkam T (2020). Acute dapagliflozin administration exerts cardioprotective effects in rats with cardiac ischemia/reperfusion injury. *Cardiovascular Diabetology*.

[33] Lee T, Chang N, Lin S (2017). Dapagliflozin, a selective SGLT2 Inhibitor, attenuated cardiac fibrosis by regulating the macrophage polarization via STAT3 signaling in infarcted rat hearts. *Free Radical Biology and Medicine*.

[34] Paolisso P, Bergamaschi L, Santulli G, Gallinoro E, Cesaro A, Gragnano F (2022). Infarct size, inflammatory burden, and admission hyperglycemia in diabetic patients with acute myocardial infarction treated with SGLT2-inhibitors: a multicenter international registry. *Cardiovascular Diabetology*.

[35] The Lancet Diabetes & Endocrinology (2015). Getting to the heart of the matter in type 2 diabetes. *The Lancet Diabetes & Endocrinology*.

[36] Ceriello A, Genovese S, Mannucci E, Gronda E (2015). Understanding EMPA-REG OUTCOME. *The Lancet Diabetes & Endocrinology*.

[37] Gilbert RE, Connelly KA (2015). Understanding EMPA-REG OUTCOME. *The Lancet Diabetes & Endocrinology*.

[38] Curtain JP, Docherty KF, Jhund PS, Petrie MC, Inzucchi SE, Køber L (2021). Effect of dapagliflozin on ventricular arrhythmias, resuscitated cardiac arrest, or sudden death in DAPA-HF. *European Heart Journal*.

[39] Zelniker TA, Bonaca MP, Furtado RHM, Mosenzon O, Kuder JF, Murphy SA (2020). Effect of Dapagliflozin on Atrial Fibrillation in Patients with Type 2 Diabetes Mellitus: Insights From the DECLARE-TIMI 58 Trial. *Circulation*.

[40] Bonora BM, Raschi E, Avogaro A, Fadini GP (2021). SGLT-2 inhibitors and atrial fibrillation in the Food and Drug Administration adverse event reporting system. *Cardiovascular Diabetology*.

[41] Fernandes GC, Fernandes A, Cardoso R, Penalver J, Knijnik L, Mitrani RD (2021). Association of SGLT2 inhibitors with arrhythmias and sudden cardiac death in patients with type 2 diabetes or heart failure: a meta-analysis of 34 randomized controlled trials. *Heart Rhythm*.

[42] von Lewinski D, Tripolt NJ, Sourij H, Pferschy PN, Oulhaj A, Alber H (2022). Ertugliflozin to reduce arrhythmic burden in ICD/CRT patients (ERASe-trial) – a phase III study. *American Heart Journal*.

[43] Zelniker TA, Wiviott SD, Raz I, Im K, Goodrich EL, Bonaca MP (2019). SGLT2 inhibitors for primary and secondary prevention of cardiovascular and renal outcomes in type 2 diabetes: a systematic review and meta-analysis of cardiovascular outcome trials. *Lancet*.

[44] Palmer SC, Tendal B, Mustafa RA, Vandvik PO, Li S, Hao Q (2021). Sodium-glucose cotransporter protein-2 (SGLT-2) inhibitors and glucagon-like peptide-1 (GLP-1) receptor agonists for type 2 diabetes: systematic review and network meta-analysis of randomised controlled trials. *BMJ*.

[45] Tripolt NJ, Kolesnik E, Pferschy PN, Verheyen N, Ablasser K, Sailer S (2020). Impact of EMpagliflozin on cardiac function and biomarkers of heart failure in patients with acute MYocardial infarction—the EMMY trial. *American Heart Journal*.

[46] Sulo G, Sulo E, Jørgensen T, Linnenberg A, Prescott E, Tell GS (2020). Ischemic heart failure as a complication of incident acute myocardial infarction: Timing and time trends: a national analysis including 78,814 Danish patients during 2000–2009. *Scandinavian Journal of Public Health*.

[47] Heidenreich PA, Bozkurt B, Aguilar D, Allen LA, Byun JJ, Colvin MM (2022). 2022 AHA/ACC/HFSA Guideline for the Management of Heart Failure: A Report of the American College of Cardiology/American Heart Association Joint Committee on Clinical Practice Guidelines. *Circulation*.

[48] Wolsk E, Claggett B, Pfeffer MA, Diaz R, Dickstein K, Gerstein HC (2017). Role of B‐Type Natriuretic Peptide and N‐Terminal Prohormone BNP as Predictors of Cardiovascular Morbidity and Mortality in Patients with a Recent Coronary Event and Type 2 Diabetes Mellitus. *Journal of the American Heart Association*.

[49] Lee J, Choi E, Khanam SS, Son J, Youn Y, Ahn M (2019). Prognostic value of short-term follow-up B-type natriuretic peptide levels after hospital discharge in patients with acute myocardial infarction. *International Journal of Cardiology*.

[50] Morrow DA (2005). Prognostic Value of Serial B-Type Natriuretic Peptide Testing during Follow-up of Patients with Unstable Coronary Artery Disease. *JAMA*.

[51] Januzzi JL, Xu J, Li J, Shaw W, Oh R, Pfeifer M (2020). Effects of Canagliflozin on Amino-Terminal Pro–B-Type Natriuretic Peptide: Implications for Cardiovascular Risk Reduction. *Journal of the American College of Cardiology*.

[52] Januzzi JL, Zannad F, Anker SD, Butler J, Filippatos G, Pocock SJ (2021). Prognostic Importance of NT-proBNP and Effect of Empagliflozin in the EMPEROR-Reduced Trial. *Journal of the American College of Cardiology*.

[53] Verma S, Mazer CD, Yan AT, Mason T, Garg V, Teoh H (2019). Effect of Empagliflozin on Left Ventricular Mass in Patients with Type 2 Diabetes Mellitus and Coronary Artery Disease: The EMPA-HEART CardioLink-6 Randomized Clinical Trial. *Circulation*.

[54] Damman K, Beusekamp JC, Boorsma EM, Swart HP, Smilde TDJ, Elvan A (2020). Randomized, double‐blind, placebo‐controlled, multicentre pilot study on the effects of empagliflozin on clinical outcomes in patients with acute decompensated heart failure (EMPA‐RESPONSE‐AHF). *European Journal of Heart Failure*.

[55] Anker SD, Butler J, Filippatos G, Ferreira JP, Bocchi E, Böhm M (2021). Empagliflozin in Heart Failure with a Preserved Ejection Fraction. *New England Journal of Medicine*.

[56] Januzzi JL, Butler J, Zannad F, Filippatos G, Ferreira JP, Pocock SJ (2022). Prognostic Implications of N-Terminal Pro–B-Type Natriuretic Peptide and High-Sensitivity Cardiac Troponin T in EMPEROR-Preserved. *JACC: Heart Failure*.

[57] Chambergo-Michilot D, Tauma-Arrué A, Loli-Guevara S (2021). Effects and safety of SGLT2 inhibitors compared to placebo in patients with heart failure: a systematic review and meta-analysis. *IJC Heart & Vasculature*.

[58] Zhou H, Peng W, Li F, Wang Y, Wang B, Ding Y (2022). Effect of Sodium-Glucose Cotransporter 2 Inhibitors for Heart Failure With Preserved Ejection Fraction: A Systematic Review and Meta-Analysis of Randomized Clinical Trials. *Frontiers in Cardiovascular Medicine*.

[59] Solomon SD, McMurray JJV, Claggett B, de Boer RA, DeMets D, Hernandez AF (2022). Dapagliflozin in Heart Failure with Mildly Reduced or Preserved Ejection Fraction. *New England Journal of Medicine*.

[60] Jhund PS, Kondo T, Butt JH, Docherty KF, Claggett BL, Desai AS (2022). Dapagliflozin across the range of ejection fraction in patients with heart failure: a patient-level, pooled meta-analysis of DAPA-HF and DELIVER. *Nature Medicine*.

[61] de Koning MLY, Westenbrink BD, Assa S, Garcia E, Connelly MA, van Veldhuisen DJ (2021). Association of Circulating Ketone Bodies with Functional Outcomes after ST-Segment Elevation Myocardial Infarction. *Journal of the American College of Cardiology*.

[62] Baker HE, Kiel AM, Luebbe ST, Simon BR, Earl CC, Regmi A (2019). Inhibition of sodium–glucose cotransporter-2 preserves cardiac function during regional myocardial ischemia independent of alterations in myocardial substrate utilization. *Basic Research in Cardiology*.

[63] Voorrips SN, Boorsma EM, Beusekamp JC, De-Boer RA, Connelly MA, Dullaart RPF (2022). Longitudinal Changes in Circulating Ketone Body Levels in Patients with Acute Heart Failure: a Post Hoc Analysis of the EMPA-Response-AHF Trial. *Journal of Cardiac Failure*.

[64] Ferrannini E, Baldi S, Frascerra S, Astiarraga B, Heise T, Bizzotto R (2016). Shift to Fatty Substrate Utilization in Response to Sodium–Glucose Cotransporter 2 Inhibition in Subjects without Diabetes and Patients with Type 2 Diabetes. *Diabetes*.

[65] Packer M, Anker SD, Butler J, Filippatos G, Pocock SJ, Carson P (2020). Cardiovascular and Renal Outcomes with Empagliflozin in Heart Failure. *New England Journal of Medicine*.

[66] McMurray JJV, Solomon SD, Inzucchi SE, Køber L, Kosiborod MN, Martinez FA (2019). Dapagliflozin in Patients with Heart Failure and Reduced Ejection Fraction. *New England Journal of Medicine*.

[67] Neal B, Perkovic V, Mahaffey KW, de Zeeuw D, Fulcher G, Erondu N (2017). Canagliflozin and Cardiovascular and Renal Events in Type 2 Diabetes. *New England Journal of Medicine*.

[68] Chew DS, Heikki H, Schmidt G, Kavanagh KM, Dommasch M, Bloch Thomsen PE (2018). Change in Left Ventricular Ejection Fraction Following first Myocardial Infarction and Outcome. *JACC: Clinical Electrophysiology*.

[69] Wu WY, Biery DW, Singh A, Divakaran S, Berman AN, Ayuba G (2020). Recovery of Left Ventricular Systolic Function and Clinical Outcomes in Young Adults With Myocardial Infarction. *Journal of the American College of Cardiology*.

